# Double Haploid Development and Assessment of Androgenic Competence of Balkan Pepper Core Collection in Bulgaria

**DOI:** 10.3390/plants10112414

**Published:** 2021-11-09

**Authors:** Stanislava Grozeva, Gancho Pasev, Vesela Radeva-Ivanova, Velichka Todorova, Valentina Ivanova, Amol N. Nankar

**Affiliations:** 1 Maritsa Vegetable Crops Research Institute (MVCRI), 4003 Plovdiv, Bulgaria; stanislava_grozeva@abv.bg (S.G.); gipasev@aol.com (G.P.); vesela_radeva@izk-maritsa.org (V.R.-I.); todorova_vili@abv.bg (V.T.); 2 Center of Plant Systems Biology and Biotechnology (CPSBB), 4000 Plovdiv, Bulgaria; vivanova@cpsbb.eu

**Keywords:** androgenesis, pepper varietal groups, Bulgarian peppers, haploid: diploid regenerants, PMMoV resistance, capsicum double haploids

## Abstract

This study was designed to assess the androgenic potential of 180 pepper accessions and 11 progenies (four F1 and seven BC) possessing PMMoV resistance in order to complement an ongoing pepper breeding program. The experiment was carried out in 10 replications with 20 anthers for each accession in two different induction mediums from 2017 to 2019. The highest androgenic response was observed in culture medium 17-2 but differences between two mediums were nonsignificant. From a total of 191 genotypes, 102 genotypes expressed a potential for direct embryogenesis. Embryo induction was seen to be genotype-dependent and decreased in the following order: Pumpkin > Conical > Bell or blocky > Round > Elongate as the most responsive genotypes with over 10% reacted anthers being observed in CAPS-23, CAPS-29, CAPS-127, CAPS-157, CAPS-169, F1 and BC 887 derived from CAPS-23. The number of regenerated plants was higher in the conical group and least in the round varietal group. Regenerated plants were examined visually and by flow cytometry for identification of spontaneous doubled haploids (DH) and haploids. Those originating from F1 and BC progenies were additionally evaluated by a CAPS marker targeting L4 allele for resistance against PMMoV. Obtained results revealed two groups consisting of homozygous susceptible and resistant plants. Therefore, use of anther culture in ongoing breeding will greatly facilitate the pepper genetic improvement.

## 1. Introduction

Pepper is a widespread vegetable well known for its diverse uses in food and spice, and as an ornamental and medicinal plant across the world [1]. Pepper has a significant economic and socio-cultural importance across the Balkan peninsula and has appreciable diversity comprised of unique forms that are not grown in other parts of the world [2,3]. Within the Balkans, Bulgaria has a congenial climate that favors pepper production and farmers have been encouraged to produce peppers for targeted niche markets [4]. The region-specific and large genetic diversity of local forms, well adapted in the Balkan peninsula, have been used to develop new varieties and F1 hybrids by trait introgression in existing cultivars [5,6]. On one hand, loss of genetic material is oftenly connected with the high rate of cross-pollination in pepper [7], while on the other hand varieties developed by conventional breeding require prolonged duration and abundant resource; hence, utilization of other crop improvement tools is critical to achieve genetic gain.

Haploidy is an important tool in plant breeding that increases the possibly to utilize the whole spectrum of genetic diversity. Use of chromosome doubling (either spontaneous or induced) allows completely homozygous plants with unique genetic recombinants to be obtained in a single generation [7,8,9], thus expediting the breeding process. The frequency of obtaining spontaneous haploids naturally in agricultural crops is very low (10^−6^ to 10^−3^). Therefore, different methods for haploid induction including parthenogenesis, interspecific hybridization, and pollination with irradiated pollen have been exploited [10,11]. In addition to these techniques, in vitro androgenesis via anther or microspore culture have been seen as very viable and successful techniques to obtain haploids and doubled-haploids in pepper [12,13,14].

Application of anther culture in pepper dates back to early 1970s [15,16,17,18,19]. Numerous studies unambiguously demonstrated that the ability of embryo induction in pepper anther culture from immature pollen and subsequent conversion into plants highly depends on genotype, age of donor plant, microspore developed stage, pretreatments, and composition of nutrient media [20,21,22,23,24]. Amongst all factors that influence embryo induction, genotype is the most important and oftenly critical for successful anther culture development. Due to its specific impact on androgenic potential, some authors have pointed out that genetic constitution dominates over the cultivation conditions [22]. Optimized growing conditions of donor plant or culture media composition does not eliminate the genotype influence but could positively affect the frequency of anther-derived embryos and their subsequent development into normal plants [25,26]. Additionally, recurrent issue of the ‘‘recalcitrant’’ nature of some pepper genotypes can be addressed if hybridization is done with responsive genotypes that can transfer the genes that determine embryogenic reaction. This approach could be vital for genotypes that possess good agricultural traits but are unresponsive to embryogenesis.

Resistance to viruses is one of the important criteria for the quality and quantity of pepper production. Tobamoviruses, particularly mechanically transmitted pathogens, are a serious threat. Major representatives include tobacco mosaic virus (TMV), tomato mosaic virus (ToMV), paprika mild mottle virus (PaMMV), and pepper mild mottle virus (PMMoV). Tobamoviruses are grouped in four pathotypes (P0, P1, P1.2, and P1.2.3), resistance to which is controlled by L1, L2, L3, and L4 allelic genes, respectively [27,28]. The L4 allele is the most suitable among these since it has a broad spectrum and provides resistance to these four pathotypes. Currently, few molecular markers are available for tracing the L4 allele [29,30,31]. Incorporation of resistance alleles in new germplasm with valuable traits is of tremendous importance for breeders. This important step allows the release of stable lines as prebreeding material. The coalesced approach of combining androgenesis-derived DH production and application of molecular marker techniques seems feasible to achieve these goals [32].

The aim of this work was to determine the androgenic response of 180 pepper accessions of Balkan origin and inculcate androgenesis to generate doubled haploid lines resistant to PMMoV using F1 and backcross (BC) heterozygous progenies from the most responsive genotypes. Development of a pipeline for releasing DH resistant lines is of substantial importance for producing lines that are suitable for F1 hybrid development in the existing pepper breeding program.

## 2. Results

### 2.1. Assessment of Androgenic Competence

**Figure 1:**
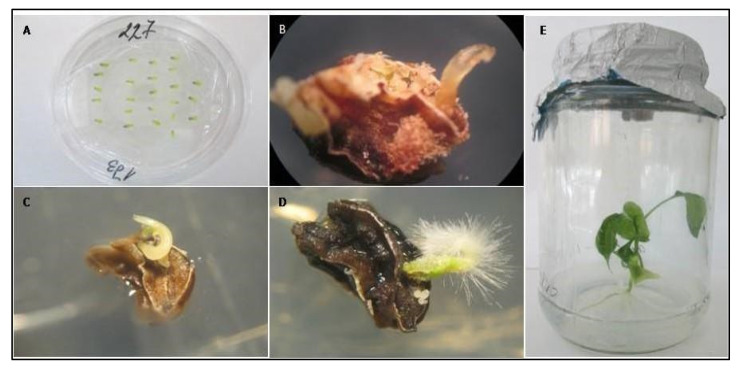
In vitro anther culture and plant regeneration. (**A**) Anther cultivation. (**B**) Induction of embryo formation. (**C**) Embryo development. (**D**) Embryo conversion. (**E**) Plant-regenerants.

**Figure 2:**
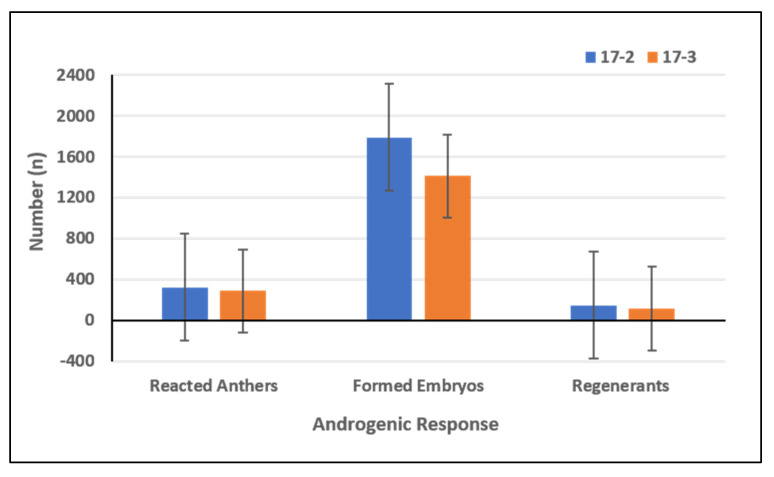
Effect of induction medium variants 17-2 and 17-3 on androgenic response of 180 pepper accessions.

The data presented in Table S1 shows that not all accessions under investigation were able to produce embryos. From a total of 180 pepper accessions, 91 expressed a potential for direct embryogenesis (Table S1). Summarized results showed the embryogenic reaction in 613 (0.85%) cultivated anthers and 3201 formed embryos (4.45%) (Table 1). From these structures 265 normal plants were regenerated. Therefore, the average conversion ratio of total embryos to normal developed plants was 8.28%. Embryo induction in anther culture showed high genotype dependency. In terms of reacted anthers and formed embryos, the accessions CAPS-23, CAPS-29, CAPS-127, CAPS-157 and CAPS-169 were the most responsive genotypes with over 40 reacted anthers and 200 to 369 formed embryos (Figure 1 and Table S1). A formed embryo with a frequency over 25% was established in four accessions (CAPS-104, CAPS-105, CAPS-128, and CAPS-164) and from 15% to 21% in three accessions (CAPS-38, CAPS-108, and CAPS-165) (Table S1). According to the classification of Mityko et al. [33], four accessions displayed fair androgenic response (CAPS-23, CAPS-127, CAPS-157, and CAPS-169) while 49 accessions had poor androgenic response and 127 accessions did not develop any regenerants (Table S1). The highest number of plant regenerants was registered in accession CAPS-23 (30 plants), followed by accessions CAPS-169 (24 plants), CAPS-127 (21 plants), and CAPS-157 (21 plants) (Table S1). However, the highest conversion ratio of total embryos to plant-regenerants was found in CAPS-24 (41.03%) and CAPS-90 (39.13%) (Table S1). A conversion ratio over 20% was registered in accessions CAPS- 36, CAPS-39, CAPS-41, CAPS-92 and CAPS-159 (Table S1). A lack of further development of the obtained embryos to plants was found in 38 accessions reacted with direct embryogenesis (Table S1). Induction of microspore embryogenesis was found in both culture medium variants (17-2 and 17-3) (Figure 2). The number of reacted anthers, formed embryos and plant-regenerants was highest in variant 17-2.

**Figure 3:**
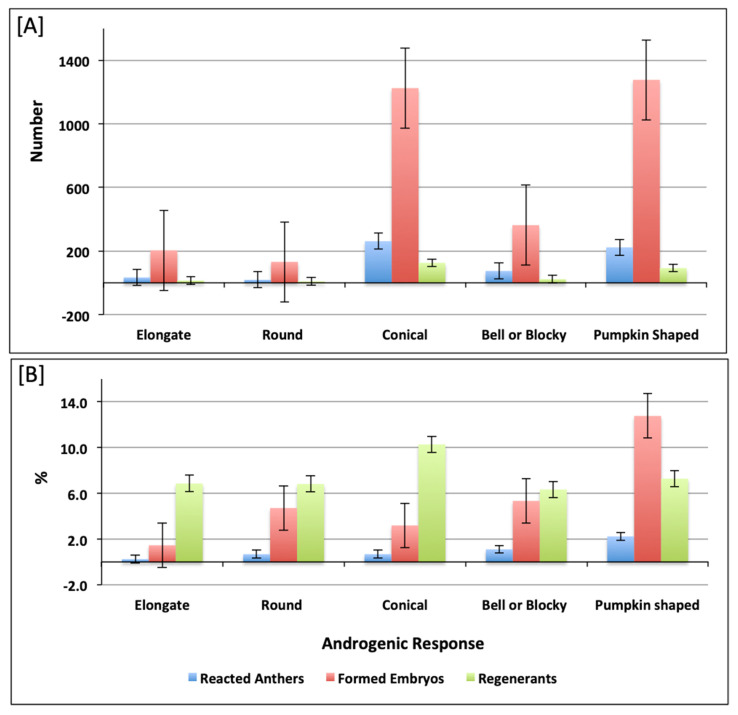
Histogram depicting reacted anthers, formed embryos, and embryo regenerants in accessions belonging to respective varietal groups. The reacted anthers, formed embryos, and embryo regenerants measured in number and percentage (%) are presented in (**A**,**B**), respectively.

**Figure 4:**
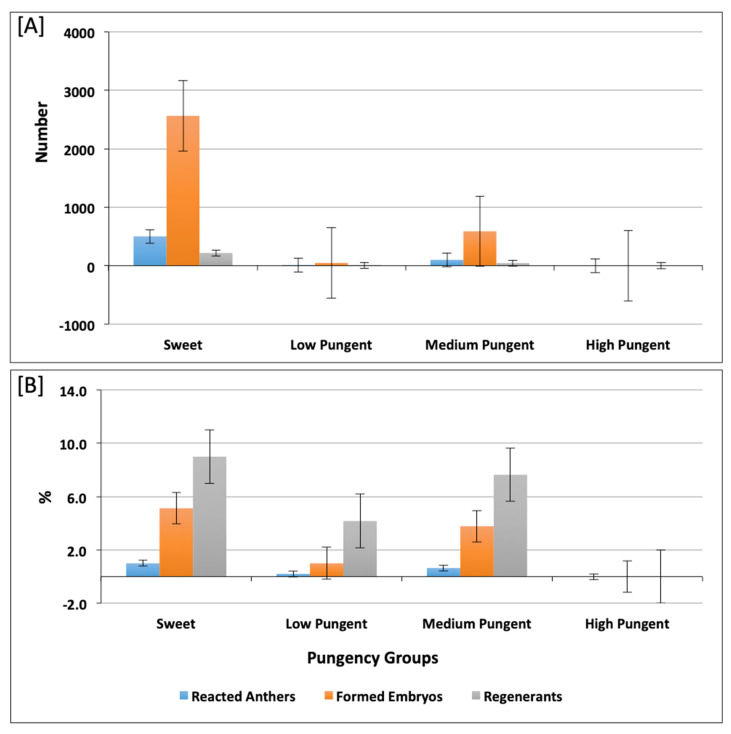
Histogram depicting reacted anthers, formed embryos, and embryo regenerants in accessions belonging to sweet, low pungent, medium pungent, and high pungent groups. The reacted anthers, formed embryos, and embryo regenerants measured in number and percentage (%) are shown in (**A**,**B**), respectively.

In this investigation, a broad range of pepper accessions belonging to five different predefined pepper varietal groups was included. The androgenic response of the studied accessions decreased from Pumpkin > Conical > Bell or Blocky > Round > Elongate. The number of regenerated plants was higher in Conical accessions (126 plants) and lower in Elongate and Round, with fourteen and nine plants, respectively (Table S2 and Figure 3A). The highest androgenic potential was obtained in accessions from the Pumpkin-shaped varietal group with 2.23% reacted anthers and 12.77% formed embryos (Figure 3B). Consideraing the varietal group diversity in the investigated pepper collection, we aimed to assess the differences between the varietal groups for the measured androgenic responses in both culture medium 17-2 and 17-3. We observed that there were no significant differences between varietal groups in both culture medium 17-2 (Table S3A) and 17-3 (Table S3B). In most cases, the pungent accessions showed a weaker reaction or did not form embryos at all, as shown in Figure 4A, for androgenic response measured in numbers, a Figure 4B for androgenic response measured in percentage (%). We also made multiple comparisons between accessions possessing varied pungency and androgenic response of accessions belonging to different pungent groups (as shown in Table S4A,B) for culture medium 17-2 and 17-3, respectively.

Data presented in Table 2 show that the hybrid combinations possessed better androgenic responses than their respective parents, except for CAPS-23. The results also show that the hybrid combinations derived from CAPS-19 and CAPS-23 were more efficient in direct embryogenic induction compared to hybrids derived from CAPS-30 and CAPS-87. Plant regenerant number was greatest in hybrid combination with CAPS-23 followed by CAPS-19. A significantly lower number of obtained regenerants was reported in hybrid combinations of CAPS-30 and CAPS-87.

The same tendency was observed in BC progeny. Lines 887 and 886 were seen as the most responsive BC progenies among those tested, in which 22% and 17%, respectively, of cultured anthers succeeded to form embryos. These two genotypes had the best results in the number of regenerated plantlets per bud. In terms of the percentage of embryo conversion to plants, this was highest in lines 782 and 882 (9.09% and 9.80%, respectively).

### 2.2. Discrimination of Haploid and Doubled Haploid Regenerants

**Figure 5:**
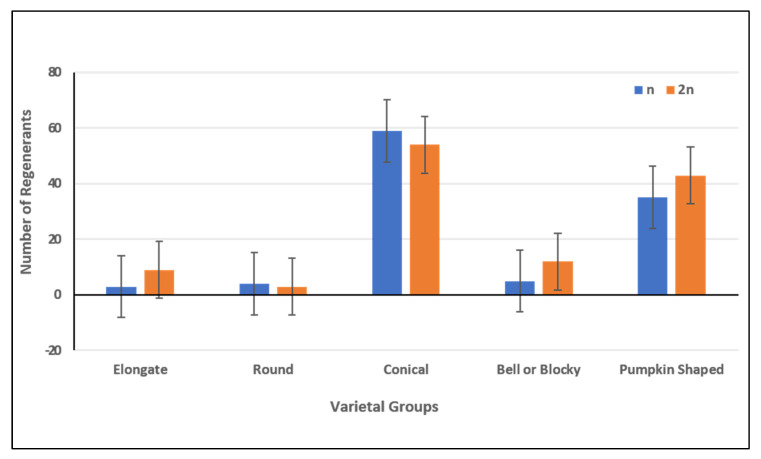
Number of plant-regenerants with different haploid and diploid chromosome levels.

A few approaches were employed for discrimination between haploid and doubled haploid regenerants. Flow cytometric analysis of plants showed haploid chromosome sets in 46.7% and diploid sets in 53.3% of the plants. The ratio of haploid:diploid plants was 1: 1.1 (Figure 5). With regard to the varietal groups, the ratio was aproximately 1:3 in Elongate accessions, 1.1:1 in Round and Conical, 1:2.4 in Bell or blocky and 1:1.2 in Pumpkin-shaped accessions.

**Figure 6:**
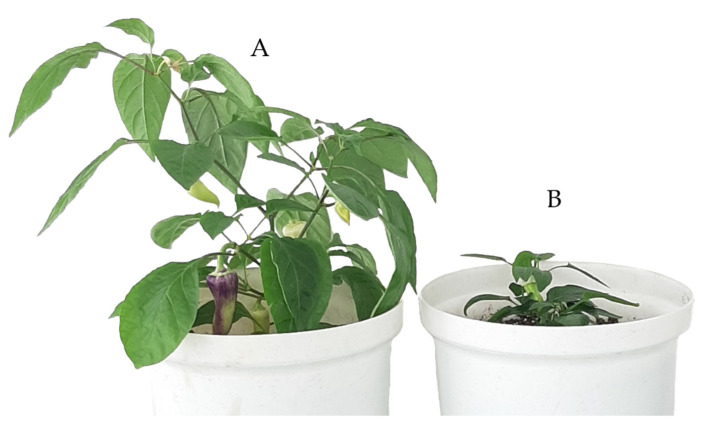
Morphological differences between doubled-haploid (**A**) and haploid (**B**) plants.

Three doubled haploid and five haploid regenerants were used for counting the number of chloroplasts in stomata cells. In haploids, the number of chloroplast grains was between four to six per cell for all tested varietal types, while for doubled haploid individuals this number ranged between seven to nine per cell. Visual observation of plant habitus and leaf size were not always informative regarding the chromosome sets. In some cases, regenerants with dwarf habitus were able to produce seeds, which was one of the best criteria indicating individuals with two identical chromosome sets (Figure 6).

### 2.3. Identification of Resistance in Doubled Haploid Individuals by Polymerase Chain Reaction (PCR)

**Figure 7:**
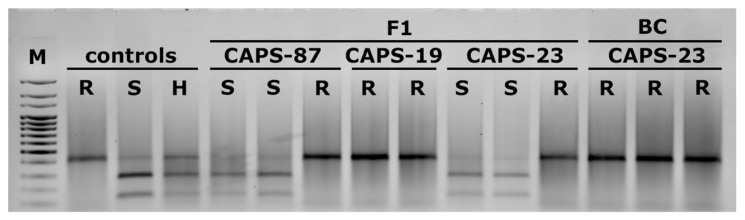
Restriction pattern of CAPS marker for identification of the L4 allele conferring resistance to PMMoV in F1 and BC regenerants. Controls represent diploid genotypes. R—resistant (L4L4), S—susceptible (L0L0), H—heterozygous (L4L0); M—100 bp marker.

PCR analysis of DH pepper plants originating from F1 and BC progenies identified individuals possessing the resistant L4 or susceptible L0 allele in homozygous conditions (Figure 7). The L4 allele was found in regenerants from the F1 generation developed with CAPS-87, CAPS-19 and CAPS-23 as elite components. Analysis of haploid plants possessing the L4 or L0 allele showed the same restriction profile as doubled haploid individuals possessing L4 or L0, respectively. No pattern representing both alleles in a single genotype was found. Similarly, regenerants with L alleles were obtained from BC progenies, but they originated from CAPS-23 only (Figure 7).

## 3. Discussion

Anther culture is one of the most used techniques in pepper as a fast and efficient way to obtain doubled haploid plants. However, microspore embryogenesis induction by anther culture has only been achieved in a reduced number of genotypes. Several investigations have been conducted on the development of successful protocols related to pepper anther culture, but androgenic efficiency has been seen to be genotype-dependent [22,25,34,35,36,37]. The effect of genotype on androgenesis of 180 pepper accessions and four F1 hybrids was investigated in the present study. Among accessions, significant differences in androgenic response were observed ranging from 0.0% to 12.0%. The number of obtained regenerants was 265, with a conversion ratio of 8.28%. Olszewska et al. [26] obtained 46 plants from 115 embryos (40.0%) and reported androgenic efficiency in 17 capsicum genotypes from 0% to 6.15%. Niklas-Nowak et al. [38] evaluated individual plant reactions of the *C. annuum* hybrid (‘ATZ1’ × ‘TG’) F2, interspecific hybrid (*C. frutescens* × *C. chinense*) F2 and the androgenic DH line AT6 in anther culture. Clear differences in androgenic effectiveness were demonstrated among the individual pepper plants. After studying androgenic response of more than 500 varieties, breeding lines and F1 hybrids, Mityko and Fari [39] found reduction of androgenic potential in the order of wax type > dark green type > tomato shape > hot pepper (from 76 to 0 plants per 100 anthers). Our results indicated that embryogenic efficiency decreased from Pumpkin > Conical > Bell or blocky > Round > Elongate. This finding is similar to those reported by Koleva-Gudeva et al. [40] where bell-shape and sweet pepper genotypes had higher androgenic potential than the hot genotypes. The authors assumed that capsaicin has inhibitory activity on haploid embryo formation in pepper. Pelliccione [41] also concluded that the hot pepper genotypes were less responsive than the sweet pepper genotypes. Moreover, it was found that the anthers of hot genotypes suffered considerable oxidation during culture, which may have caused low androgenic response among these genotypes.

**Figure 8:**
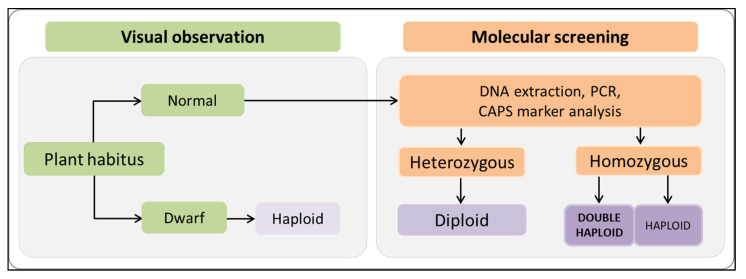
Combined approach of visual observation and molecular screening to discriminate haploid, doubled haploid, and diploid androgenic individuals. Adopted plants were grown in soil mixture and were inspected visually for dwarf habitus. In second phase, normal individuals were subjected to molecular analysis by a CAPS marker to discriminate zygotic state of L4 allele for PMMoV resistance.

The results in this study showed better androgenic frequency in the hybrids compared to their respective parents. According to Mityko et al. [39], F1 hybrids produced from crosses between poor/non-responsive and responsive genotypes showed a fair level of response, even in the case of a poor response in the donor parent. We also observed a difference in the androgenic response between F1 and BC progenies. Higher values were recorded for BC lines. We consider the difference is due to the different amount of genetic material of CAPS-23 in both progeny types. The CAPS-23 component in F1 is 50% and 75% in BC. It seems that the good androgenic potential of CAPS-23 serves as a booster by recovering its genome in the BC progenies. Substantial variation was also observed within the BC group. We can only speculate that this might be due to recombination events that interfere the androgenic potential. Discrimination between haploid and diploid plants is one of the pressing challenges widely reported by anther culture researchers. However, we chose a combined approach of visual observation and molecular analysis to help us resolve this issue. It is well known that generally haploid plants develop dwarf habitus that is easy to recognize. However, the main challenge appears to be in distinguishing doubled haploid from the diploid plants. In our case, somatic cells of all F1 individuals possessed a combination of L4 and L0 alleles. Thus, regenerants originating from somatic tissues were expected to possess both alleles. On the other hand, the male gametophyte was expected to produce two types of pollen grains, L4 and L0. Every plant originating from a pollen grain only adopted one of the alleles. Thus, discrimination of DH and diploid was easily done by the CAPS marker 087H3T7, which characterizes the zygotic state of the L4 allele (Figure 8) and is useful for selection of PMMoV resistant lines. The flow cytometric procedures provided information only for chromosome number i.e., n and 2n. This information was insufficient in discriminating the nature of 2n. Hence our discrimination model using a CAPS marker was much easier and informative, and can be adopted for other doubled haploid programs once a suitable marker is available. A similar approach using a SSR codominant marker was utilized by Keles et al. [13], who were able to determine whether the spontaneous doubled haploid plants were homozygous. In other reports, molecular markers were used to estimate the level of homogeneity versus heterogeneity by comparison with control anther donor by RAPD, SSR, and ISSR-PCR markers [42,43]. Caranta et al. [44] studied 138 markers, mostly RFLPs and RAPDs, for characterization of 94 DH lines originating from a cross between Perennial and Yolo Wonder for resistance to cucumber mosaic virus (CMV). Authors identified that genotypic variance among the DH lines was highly significant for the number of local lesions caused by CMV, and heritability was estimated to be 0.94.

## 4. Materials and Methods

### 4.1. Plant Material

**Figure 9:**
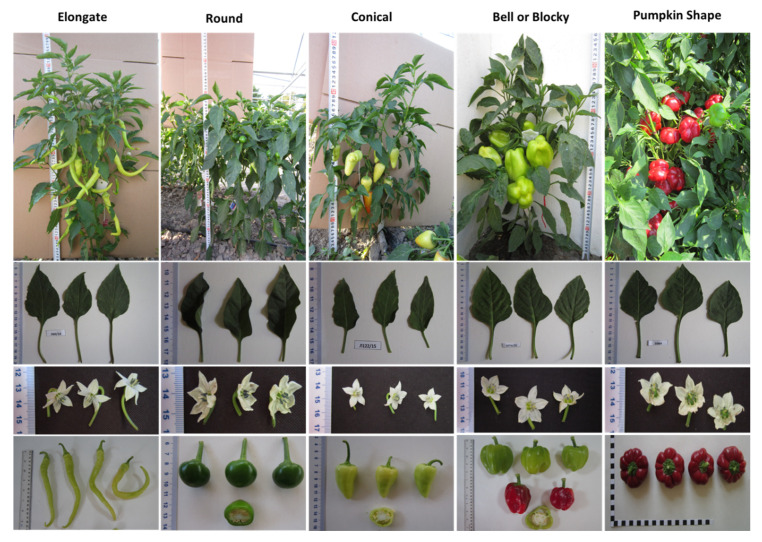
Varietal groups representing morphological variation of leaf, flower, fruit, and plant habitus. Accessions CAPS-10, CAPS-2, CAPS-7, CAPS-29, and CAPS-38 represent the Elongate, Round, Conical, Bell or Blocky, and Pumpkin-shaped varietal groups, respectively.

The experimental work was conducted with 180 pepper accessions of *Capsicum annuum* L. belonging to Elongate (35), Round (7), Conical (96), Bell or blocky (17), and Pumpkin shaped (25) varietal groups. This pepper collection was created a decade ago based on expeditions, national and international projects and germplasm exchange and personal contacts. It was collected from six different Balkan countries including Bulgaria (114), Serbia (28), Macedonia (16), Romania (9), Albania (7), and Greece (3), with three accessions of unknown origin, although the majority of the accessions are from Bulgaria. Hitherto, these accessions have been characterized for plant architecture, fruit shape, color, taste, biological value and consumption of the fruits, and represent Balkan capsicum diversity. More information about these accessions, their origin and their agro-morphological features, have been published in previous studies [3,4]. The details about each varietal group and their description are shown in Table 3 and Figure 9. Eleven progenies (four F1 and 7 BC) resulted from crosses of four elite Bulgarian cultivars and a line resistant to PMMoV (R21). Cultivars, namely, Ivaylovska kapia (CAPS-87), Stryama (CAPS-23), Sivria 600 (CAPS-19), and Byal kalinkov (CAPS-30) are well-adopted local varieties and possess enhanced nutritional quality. The resistant line R21 was created by self-pollination of individual plant from a segregating kapia-type population possessing the L4L4 resistant genotype identified by the 087H3T7 marker (see Section 4.4 and Section 4.5). The BC accessions were developed by back-crossing F1 (CAPS-23 × R21) plants with the elite parent CAPS-23 as the male component (F1 × CAPS-23). Further, only heterozygous BC individuals were selected by a detached leaf test (DLT) method [3], while homozygous susceptible plants were discarded. Plants were grown in 5 L pots filled with peat moss and perlite mixture in a ratio of 1:1 (*v*/*v*) in a glasshouse under natural light conditions and temperature ranging from 15 °C to 30 °C during the period April to October 2017 to 2019.

### 4.2. Anther Culture

Anthers with pollen in late mononuclear and early binuclear stages (flower bud lengthwise 3.5–4.0 mm) were excised after surface sterilization in 5% calcium hypochlorite and rinsed in sterile distilled water [45]. The anthers were isolated and cultivated in Petri dishes on two variants of induction medium containing micro and macrosalts by Murashige & Skoog [46], vitamins by Gamborg et al. [47] (Duchefa Biochemie, Netherlands) (MS0), 0.1 mgL^−1^ Kinetin, 0.005 mgL^−1^ Biotin, 0.1 mgL^−1^ Glycine, 0.04 mgL^−1^ Vitamin B12, 30 gL^−1^ sucrose, and 0.7% agar different in their concentrations of 2,4-Dichlorophenoxyacetic acid (2,4-D):

- 17-2–0.1 mgL^−1^ 2,4-D
- 17-3–0.3 mgL^−1^ 2,4-D

The anthers were treated in darkness at 35 ± 1 °C for the first 8 days followed by transfer to 26 ± 1 °C at a photosynthetic proton flux density (PPFD) of 200 μmol m^−2^ s^−1^ with a 14/10 h photoperiod. After 12 days the anthers were incubated on the same medium without plant growth regulators [18]. When the regenerants were 2 cm long, they were transferred into 250 mL glass vessels containing 25 mL of rooting media comprised of ½ MS0 medium, 30 gL^−1^ sucrose, and 0.7% agar, and were incubated in a growth chamber. All culture media was adjusted to pH = 5.8 before being autoclaved, then autoclaved at 121 °C for 20 min.

In the course of experiments, the relative numbers of reacted anthers (to the in vitro cultivated anthers), the frequency of embryo formation (to the in vitro set initial explants), and the relative number of obtained plant-regenerants (to the in vitro set initial explants and to the obtained embryos) were calculated as percentages. The androgenic potential of the accessions was determined as in the Mityko et al. [33] classification. The experiment was carried out with 10 replications each with 20 anthers for different genotypes and induction medium variants during the period between 2017 and 2018.

### 4.3. Flow Cytometry to Analyze Ploidy Level

The ploidy level of the obtained plant regenerants was determined by flow cytometry (Partec PA-2, Görlitz, Germany). Flow cytometry analysis was performed on newly grown chopped leaves with 1 mL of nuclei extraction buffer (Solution A) filtered through a 50-lm Cell Trics filter (Partec, Germany), and mixed with DAPI staining buffer (Solution B).

### 4.4. DNA Extraction and PCR Analysis

Young leaves were collected from the pepper regenerants adopted in soil substrate. Total DNA was isolated by the CTAB (Cetyl Trimethyl Ammonium Bromide) method following the protocol described by Edwards et al. [48] with minor modifications. PCR reactions were performed in a thermocycler (Biorad T100, Feldkirchen, Germany) using a GC PCR enhancer kit (Canvax, Cordoba, Spain) in a final volume of 20 µL. The reaction mixture contained 2 µL 10x reaction buffer, 2 µL 8 mM dNTPs mix, 2.5 µL 25 mM MgCl^2^, 1.5 µL 10 mM of forward and reverse primer, 2 µL GC enhancer, 0.2 µL Horse-Power™ TaqDNA Polymerase and 1 µL template DNA. The thermal profile consisted of an initial denaturation step at 95 °C for 4 min followed by 35 cycles beginning with a denaturation step at 94 °C for 30 sec, an annealing step at 58 °C for 30 s, and an extension step at 72 °C for 1 min. The final extension was for 7 min at 72 °C to finish the synthesis of all incomplete copies of the targeted fragment.

### 4.5. CAPS Marker Resolving

Genotyping of the DH individuals was done by the codominant 087H3T7 marker [30]. The amplified fragment of 440 bp was digested with FastDigest SspI endonuclease (Thermoscientific™, Waltham, MA, USA) in a volume of 15 μL according to the manufacturer’s prescription. The products of the digestion reaction were resolved on a1%TBE agarose gel for 60 min at 80 V and visualized by SimplySafe™ (Eurx, Gdańsk, Poland).

### 4.6. Chloroplast Number Counting

To count the chloroplast number of the regenerants, the epidermis was peeled from the abaxial surface of the leaf and was placed onto a microscopic glass slide in a drop of a iodine tincture. The number of chloroplasts per guard cell was counted under light microscope from at least 10 stomata per sample.

### 4.7. Statistical Analyses

Statistical analysis was done using the *XLSTAT* program version 15. Statistical analysis included descriptive statistics, histograms, and multiple comparisons of predefined varietal groups. Multiple comparisons were done using Tukey’s test at α = 0.05 for pre-defined varietal groups and pungency groups.

## 5. Conclusions

This study elucidated that androgenesis was successfully induced in 91 accessions. Accessions belonging to the Pumpkin-shaped and Conical varietal groups showed higher androgenic potential, whereas accessions from Round and Elongate varietal groups had the least. Accessions CAPS-23, CAPS-29, CAPS-127, CAPS-157, and CAPS-169 were the most responsive in terms of reacted anthers, formed embryos, and plant regenerants. The limiting factor to androgenic response was found to be genotype. A combined approach of visual observation and use of CAPS molecular markers was more effective in identifying the doubled haploids than conventionally used visual observations. The androgenic potential of F1 progenies depended on the parent possessing such potential; however, BC progenies had higher androgenic potential compared to F1 proving the concept of the genetic background having the greatest effect. The androgenic know-how concerning the Balkan pepper core collection developed in this study will be used in the future to develop doubled haploids to complement ongoing efforts in the pepper breeding programme.

## Figures and Tables

**Table 1:** Summarized results of androgenic response in 180 accessions, 4 F1, and 7 BC pepper progenies.

Genotype	Cultivated Anthers	Reacted Anthers		Formed Embryos		Plant Regenerants		
		No.	%	No.	%	No.	%	% Towards Embryos
**Accessions**	72,000	613	0.85	3201	4.45	265	0.37	8.28
**F1**	3740	149	3.98	787	21.04	38	1.02	4.83
**BC**	1400	128	9.14	891	63.64	54	3.86	6.06

**Table 2:** Androgenic response observed in pepper F1 hybrids and their respective parents.

F1	Reacted Anthers		Formed Embryos		Plant Regenerants		
	No	%	No	%	No	%	% Toward Embryos
**F1 hybrids with**							
**CAPS-19**	13	1.76	109	14.76	13	1.76	11.93
**CAPS-23**	89	6.36	467	33.36	17	1.21	3.64
**CAPS-30**	37	6.17	165	27.50	5	0.83	3.03
**CAPS-87**	10	1.00	46	4.60	3	0.30	6.52
**Female parent**							
**CAPS-19**	0	0.00	0	0.00	0	0.00	0.00
**CAPS-23**	44	11.00	223	55.75	30	7.50	13.45
**CAPS-30**	5	1.25	38	9.50	2	0.50	5.26
**CAPS-87**	3	0.75	12	3.00	1	0.25	8.30
**BC progeny with CAPS-23**							
**782**	22	6.17	143	27.50	13	0.83	9.09
**787**	7	3.50	35	17.50	3	1.50	8.57
**788**	1	0.50	1	0.50	0	0.00	0.00
**881**	12	6.00	51	25.50	3	1.50	5.88
**882**	8	4.00	51	25.50	5	2.50	9.80
**886**	34	17.00	242	121.00	10	5.00	4.13
**887**	44	22.00	368	184.00	20	10.00	5.43

**Table 3:** Plant materials used from pepper accessions of predefined varietal groups of Elongate, Round, Conical, Bell or Blocky, and Pumpkin fruit shape. The “A” and “B” letters assigned to given accessions indicate that the selected accession differs from original population based on differences in phenotypic traits (fruit shape, colour, and taste, among others) and since then accessions have been characterised individually.

Varietal Group	Accession Number	Description
**Elongate**	CAPS-5, 8, 9, 10, 13, 14, 65, 66, 67, 69, 70, 71, 72, 73, 74, 77, 78, 71A, 109, 112, 113, 123, 124, 125, 130, 132, 137, 139, 144, 149, 153, 155, 166, 174 and 149A	Elongate fruit shape at longitudinal section and fruit length is greater than width. Most fruits are narrow at the fruit base and pointed at the blossom end.
**Round**	CAPS-1, 2, 68, 110, 115, 126 and 128	Round fruit shape at longitudinal section and fruit length is similar to width. Fruits are blunt at the blossom end.
**Conical**	CAPS-3, 6, 7, 11, 12, 15, 16, 17, 18, 19, 20, 21, 22, 23, 24, 25, 26, 27, 28, 31, 32, 39, 40, 41, 42, 43, 44, 45, 46, 47, 48, 49, 50, 51, 52, 53, 54, 55, 56, 57, 58, 59, 60,61, 62,63, 64, 75, 76, 79, 80, 81, 82, 83, 84, 85, 86, 87, 88, 89, 90, 91, 79A, 102, 108, 111, 116, 117, 118, 119, 120, 121, 122, 129, 138, 140, 141, 142, 145, 150, 151, 152, 154, 156, 157, 158, 159, 162, 168, 171, 173, 110A, 117A, 133A, 143A and 151B	Conical fruit shape at longitudinal section and fruit length is several times bigger than the width. Most fruits are pointed at the blossom end.
**Bell or Blocky**	CAPS-29, 30, 92, 93, 94, 95, 133, 134, 135, 136, 143, 146, 147, 148, 160, 161 and 121A	Rectangular or square fruit shape in longitudinal section and fruit length is almost equal to width. Most fruits have 3–5 apexes and are sunken, blunt or sunken and pointed at the blossom end.
**Pumpkin shaped**	CAPS-4, 33, 34, 35, 36, 37, 38, 96, 97, 98, 97A, 101, 103, 104, 105, 107, 114, 127, 131, 163, 164, 165, 169, 135A and 151A	Flattened fruit shape at longitudinal section and fruit length is smaller than width. Fruits are sunken or sunken and pointed at the blossom end.

## Data Availability

All the data is provided in the supplementary materials.

## Supplementary Materials

The following are available online at https://www.mdpi.com/article/10.3390/plants10112414/s1. Table S1. Androgenic response of 180 accessions. Reacted anthers, formed embryos, and plant regenerants are expressed in number and in percentage (%). Table S2. Varietal group-wise descriptive statistics of reacted anthers, formed embryos, and regenerants. Descriptive statistics is shown in mean, standard deviation (SD), and standard error (SE). Table S3. Tukey’s test for multiple comparison of varietal group for culture medium 17-2 (S3A) and 17-3 (S3B). Table S4. Tukey’s test for multiple comparison of pungency groups for culture medium 17-2 (S4A) and 17-3 (S4B).
