# Supplementary material for: Double Haploid Development and Assessment of Androgenic Competence of Balkan Pepper Core Collection in Bulgaria
Source: Plants (Basel). 2021 Nov 9;10(11):2414. doi: 10.3390/plants10112414 (PMC8625149; doi:10.3390/plants10112414)
Supplement: Supplementary file 1 [file plants-10-02414-s001.zip › Table S2. Varietal groupwise descriptive statistics (ANN, 11.08.2021).pdf]

Table S2. Varietal group wise descriptive statistics of reacted anthers, formed embryos, and regenerants. Descriptive statistics is shown in mean, standard deviation (SD), and standard error (SE).

Varietal Group	Accessions	Reacted Anthers				Formed Embryos				Regenerants			
		Total	Mean	SD	SE	Total	Mean	SD	SE	Total	Mean	SD	SE
**Elongate**	35	34	0.97	1.51	0.25	204	5.83	9.88	1.67	14	0.40	0.85	0.14
**Round**	07	19	2.71	5.12	1.94	132	18.85	40.17	15.18	09	1.28	2.98	1.13
**Conical**	96	262	2.73	7.01	0.72	1225	12.76	32.18	3.28	126	1.38	4.24	0.43
**Bell or Blocky**	17	75	4.41	11.16	2.71	363	21.35	48.20	11.69	23	1.35	3.59	0.87
**Pumpkin Shape**	25	223	8.92	13.61	2.72	1277	51.08	87.98	17.60	93	3.72	6.77	1.35
